# Comparative Analysis of Enamel Surface Integrity and Pulpal Temperature in Debonding Metallic Brackets: A Study of Two Intensity Levels of Diode Laser Versus Conventional Method

**DOI:** 10.4317/jced.62738

**Published:** 2025-06-01

**Authors:** Remmiya Mary Varghese, Ashwin George Mathew, Shreya Kishore, Lincy Rachel Thomas, Reshma Mohan

**Affiliations:** 1 Associate Professor, Department of Orthodontics and Dentofacial Orthopaedics, Saveetha Dental College & Hospitals, Saveetha Institute of Medical and Technical Sciences, Saveetha University Chennai, Tamilnadu; 2 Professor, Department of Orthodontics and Dentofacial Orthopaedics, Saveetha Dental College & Hospitals, Saveetha Institute of Medical and Technical Sciences, Saveetha University Chennai, Tamilnadu; 3 Assistant Professor, SRM Dental College, Bharathi Salai, Chennai, SRM Institute of Science and Technology, Chennai; 4 Lecturer, AIMST University, Malaysia; 5 Assistant professor, SRM Kattankulathur Dental College and Hospital,SRMIST, Chennai

## Abstract

**Background:**

The process of removing metallic orthodontic brackets creates difficulties which affect the enamel surface condition along with excessive temperature rise in the pulp. Traditional ethnomethodological debonding methods lead to enamel destruction yet the advent of diode lasers promises to decrease adverse side effects. Speakers evaluate the impact of using two different laser intensity settings during diode laser-assisted bracket debonding on enamel surface preservation andclarations of intrapulpal temperature changes.

**Material and Methods:**

A test involved sixty human premolars bonded with metallic brackets by means of a standardized adhesive system that was then randomly distributed into three groups where each group consisted of twenty premolars (Group A – conventional debonding by pliers, Group B – laser debonding at 2.5 W, and Group C – laser debonding at 3.5 W). The research used thermocouple technology to monitor pulpal temperature changes while Scanning Electron Microscopy (SEM) together with modified Adhesive Remnant Index (ARI) evaluated the condition of enamel surfaces. A statistical Analysis was performed by using ANOVA and post hoc tests in which the p value was set below 0.05.

**Results:**

The debonding technique by conventional means (Group A) generated a mean temperature elevation of 2.1 ± 0.5°C in the pulpal area while Group B with 2.5 W laser power and Group C with 3.5 W laser power led to temperature rises of 4.5 ± 0.8°C and 6.2 ± 1.1°C respectively. Quantitative evaluation through SEM revealed Group A had the highest rates of enamel damage whereas Group C registered the lowest amount of enamel modification. The ARI results confirmed that Group A experienced more adhesive residual on enamel surfaces whereas both Groups B and C maintained more adhesive material on their bracket bases during the debonding process.

**Conclusions:**

Diode laser-assisted brackets debonding conducted at low and high intensity settings produced better enamel preservation results than standard debonding methods. The pulpal temperature generated at the 3.5 W setting exceeded other measurements which justifies careful use of this method. A diode laser at 2.5 W offers a suitable tradeoff between enamel protection and pulpal thermal safety.

** Key words:**Diode laser, orthodontic debonding, enamel integrity, pulpal temperature, metallic brackets.

## Introduction

Orthodontic treatment ends by removing metallic brackets which doctors at first bonded to teeth through enamel attachment. The processes of bracket removal call for careful procedures to safeguard the enamel structure and ensure patient comfort during the treatment. Orthodontic bracket removal with mechanical pliers causes enamel fracture formations and microcrack generation and leaves excessive amounts of adhesive needing follow-up finishing and polishing treatment (1,2). Research shows that incorrect debonding forces bring about patient discomfort in addition to causing pain during the procedure (3).

Radiant diode lasers have introduced a modern technique for bracket removal. Additionally these lasers perform bracket adhesive melting to decrease the amount of mechanical force required for extraction (4). Bracket debonding efficiency gets improved when lasers assist in removal techniques since these methods minimize potential enamel damage and decrease adhesive residue reminders (5,6). The main drawback of laser usage in orthodontics stems from excessive temperature increases which can reach damaging levels above 5.5°C during procedure time (7).

Research data shows how diode laser power adjustments affect both enamel surfaces and pulpal temperature levels during bracket removal (8,9). Research data indicate that reducing laser intensity allows safe temperature control but lower intensities do not deliver the same effectiveness for adhesive softening. When using greater laser power for adhesive dislodgment the process becomes faster yet there arise safety concerns about excessive thermal damage. Pulp vitality preservation and successful retention of enamel structure during effective debonding processes are achievable when using optimal laser parameter setups (10).

The study examines how pulpal heat and enamel degradation from orthodontic bracket debonding procedure changes when different laser intensity settings are used as compared to traditional mechanical procedures. The collected research data can establish proper operational guidelines for orthogonal detaching procedures with diode lasers to ensure superior therapeutic outcomes.

An effective bracket removal technique must work to remove brackets without harming enamel damage. The regular mechanical method of bracket force removal leads to enamel damage by creating microscopic fractures and defects whenever excessive force is applied (11). Further polishing procedures are needed post-debonding because the amount of adhesive residue remaining on the enamel varies leading to possible enamel substance reduction (12). Laser-assisted bracket debonding represents a significant advancement because this technology melts the adaptive material which results in reduced bracket extraction force (13). There is no unified consensus among scientific professionals regarding optimal laser parameter settings that would deliver successful tooth bracket debonding with protection of enamel strength.

The orthodontic field approves diode lasers as work tools because they allow exact adhesive control without harming adjacent tissues (14). The laser heat reaction surpasses the maximum temperature range for pulp protection resulting in possible dental damage (15). The combination of laser diode system control factors determines how difficult brackets will separate from teeth and influences pulpal temperature elevation (16). Strong laser settings create elevated power distribution while generating more heat regardless of their ability to reduce bracket retention. Choosing an optimized laser setting is critical because it ensures correct bracket removal together with the retention of enamel integrity and protects the pulp from hazardous heat damage. A study examines the performance of two diode laser power levels when compared to standard bracket extraction methods to develop safe operational standards for dentistry.

## Material and Methods

The experimental research involved sixty human premolars which surgeons eliminated through orthodontic care. Prior to storage the teeth received cleaning procedures to remove both debris and soft tissue remainders before being submerged in 0.1% thymol solution at room temperature. The research included only human premolars that lacked caries, cracks and prior restorations.

The teeth received placements in self-cure acrylic resin blocks to reproduce clinical practices. A 30-second application of 37% phosphoric acid gel on enamel surfaces took place before performing distilled water rinsing followed by use of an oil-free stream of air for drying. The application step included light-cured orthodontic adhesive followed by placing and firmly pressing stainless steel orthodontic brackets (0.022” slot size) on the enamel surface. The excess adhesive was eliminated before LED light activation occurred for 20 seconds on each bracket surface.

The bonded teeth were randomly assigned into three groups (n=20 per group) based on the debonding technique:

• Group A (Conventional Debonding): Brackets were removed using mechanical debonding pliers by applying a controlled force.

• Group B (Diode Laser at 2.5 W): A diode laser (wavelength 980 nm, power 2.5 W, continuous mode) was used to irradiate the bracket base for 5 seconds, followed by bracket removal with pliers.

• Group C (Diode Laser at 3.5 W): A diode laser (wavelength 980 nm, power 3.5 W, continuous mode) was applied for 5 seconds, followed by mechanical removal with pliers.

The J-type thermocouple was inserted into the pulp chambers of teeth through root apex entry points using composite resin to maintain heat containment for temperature measurement. A real-time system measured the temperature variations as the laser operated.

The analysis of enamel surface integrity happened through scanning electron microscopy after debonding procedure. The clinical evaluation tool used to measure residual adhesive amount on enamel surfaces post-bracket removal was called adhesive remnant index (ARI score). The ARI scores ranged from:

• Score 0 – No adhesive remaining on the enamel.

• Score 1 – Less than 50% adhesive remaining.

• Score 2 – More than 50% adhesive remaining.

• Score 3 – Entire adhesive layer present on the bracket base.

Statistical analysis took place within SPSS software version 25.0 (IBM Corp.). One-way ANOVA was utilized to analyze pulpal temperature changes along with ARI scores across the groups while utilizing post hoc Tukey’s test to complete pairwise group examinations. An analytic significance value of less than 0.05 indicated statistical significance.

## Results

The average temperature increase inside the pulp exhibited distinct variation between the groups based on statistical analysis (*p* < 0.05). The recorded temperature elevation reached its peak with Group C (3.5 W diode laser) and then decreased in Group B (2.5 W diode laser) before Group A (conventional debonding) generated the lowest elevation. The recorded temperature increase in Groups A and B did not reach the 5.5°C critical threshold that could damage pulp tissue but Group C crossed this threshold ( Table 1).

The analysis through SEM demonstrated that enamel suffered unique damages among the tested groups. The conventional group (Group A) presented more enamel breaks as well as surface inconsistencies because of using mechanical forces. Group C that received 3.5 W diode laser treatment showed the minimum amount of enamel damage among the laser-assisted groups. The scores of Adhesive Remnant Index (ARI) showed Group A possessed the highest amount of adhesive on the enamel while Groups B and C demonstrated greater adhesive retention on bracket bases indicating decreased enamel damage ( Table 2).

The analysis using one-way ANOVA confirmed statistically relevant differences emerged among the study groups when measuring pulpal temperature rise along with enamel integrity scores (*p* < 0.05). The results from Post hoc Tukey’s test established that Group B along with Group C showed differences against Group A regarding temperature alterations and enamel maintenance levels. Group B (2.5 W diode laser) proved to be the ideal method for enamel protection through optimal pulpal temperature management during the study period.

These results suggest that diode laser-assisted debonding, particularly at 2.5 W, is a viable alternative to conventional mechanical debonding as it preserves enamel integrity while maintaining pulpal temperature within safe limits ( Table 1, Table 2). However, caution is needed when using a 3.5 W laser due to its potential to exceed the critical temperature threshold.

## Discussion

The present research evaluated the temperature changes and enamel surface damage between two diode laser intensities used for orthodontic bracket removal as well as traditional mechanical debonding approaches. The investigation shows laser-assisted debonding provides better advantages than manual procedures because it protects enamel from damage yet generates tolerable heat distributions inside the pulp. The pulpal vitality could be endangered when using laser intensity at the higher setting of 3.5 W because the heat generation reached unsafe levels.

Pulp damage leading to permanent damage may occur when the laser-assisted debonding process causes a pulpal temperature increase above 5.5°C (17). The conventional method generated the smallest increase in pulpal temperature but the 3.5 W diode laser operated at the highest level which reached above the critical threshold amount. The research data matches existing scientific reports that show laser power directly affects heat output (2,3). The thermal effects of Er:YAG and Nd:YAG laser systems confirm the requirement to control exposure times in order to stop pulpal injuries (4,5). The research conducted by Kim *et al*. (6) discovered that diode lasers with higher power levels created larger heat accumulation in enamel and dentin which matched the findings presented here.

The 2.5 W diode laser group demonstrated optimal pulpal safety and effective bracket removal because it produced a manageable temperature increase. The use of lower-power diode lasers was also effective according to Oztoprak *et al*. (18) as they reported safe pulpal temperature levels with adhesive softening. The thermal changes during therapeutic procedures require further studies regarding factors affecting this process including exposure time and distance and enamel layers (19).

The preservation of enamel stands as an essential matter when performing orthodontic debonding because overzealous mechanical forces lead to microcracks and fractures and substantial tooth surface erosion (20). SEM images showed that the practice of conventional debonding led to maximum enamel damage from the application of direct mechanical pressure. Multiple research studies have confirmed that pliers used for debonding create enamel microfractures and leave behind excessive adhesive remnants (11,12).

The superior condition of enamel occurred when using lasers for treatment where the 3.5 W setting displayed the lowest amount of enamel deterioration. Excessive heating effects of the laser on adhesive bonds let operators remove brackets using reduced force. The study findings by Kwon *et al*. (13) showed that applying laser energy instead of mechanical tools reduces enamel damage which supports this observation. ARI scores confirmed the hypothesis because the laser treatment methods left more adhesive material on bracket bases which demonstrates reduced enamel load (14,15).

The research shows that 2.5 W diode laser-assisted debonding has potential to become an acceptable surgical method for bracket removal because it protects enamel integrity while maintaining safe pulp temperature levels. Hospital workers need to deploy laser power carefully because high settings lead to thermal damage which harms pulp tissue structures. Developing suitable laser parameter sets with limited treatment times and standard cooling protocols will elevate orthodontic debonding safety (6).

Scientists must study laser-assisted debonding effects on both pulp vitality and enamel structure by performing actual tests on human subjects. The research should analyze and compare different safety and efficacy levels of Er:YAG and CO(2) laser systems to determine their optimal settings for orthogonal bracket removal procedures.

## Conclusions

Dual diode lasers applied during debonding procedures stand as an excellent replacement for standard mechanical bracket removal because they minimize enamel destruction while improving the process of explantation. With 2.5 watt diode laser devices physicians attained proper parameters that both minimized enamel damage while ensuring adequate pulp temperature safety. During operation the 3.5 W laser generated excessive heat levels that posed risks to pulp vitality. Proper parameter-level laser debonding techniques enhance clinical performance but require thermal protection measures during implementation according to the research. Additional testing on human subjects should be performed in real-life clinical settings to validate the obtained study findings.

## Figures and Tables

**Table 1:** Mean Pulpal Temperature Rise (°C) Across Different Debonding Methods.

Group	Mean Temperature Rise (°C)	Standard Deviation (±)
Group A (Conventional)	2.1	0.5
Group B (Diode Laser 2.5 W)	4.5	0.8
Group C (Diode Laser 3.5 W)	6.2	1.1

ANOVA, *p* < 0.05 (Significant difference among groups)

**Table 2:** Adhesive Remnant Index (ARI) Score Distribution.

Group	ARI Score 0 (%)	ARI Score 1 (%)	ARI Score 2 (%)	ARI Score 3 (%)
Group A (Conventional)	50%	30%	15%	5%
Group B (Diode Laser 2.5 W)	10%	25%	40%	25%
Group C (Diode Laser 3.5 W)	5%	20%	45%	30%

Higher ARI scores indicate more adhesive remaining on the bracket base and less enamel damage.

## Data Availability

The datasets used and/or analyzed during the current study are available from the corresponding author.
